# Acceptability, fidelity and implementation of systematic integrated pain management in oncology outpatient services: a process evaluation protocol for a multicentre clustered randomised pilot trial

**DOI:** 10.1136/bmjopen-2025-101935

**Published:** 2025-06-27

**Authors:** Olivia Claire Robinson, Florence Day, Elaine G Boland, Michelle Collinson, Marie Fallon, Amanda Farrin, Kate Flemming, Sean Girvan, Sue Hartup, David Meads, Adam Hurlow, Catriona Mayland, John O’Dwyer, Simon Pini, Daniel Swinson, Suzanne H Richards, Matthew R Mulvey

**Affiliations:** 1 Leeds Institute of Health Sciences, University of Leeds, Leeds, UK; 2 Clinical Trials Research Unit, University of Leeds, Leeds, UK; 3 Palliative Medicine, Hull University Teaching Hospitals NHS Trust, Cottingham, UK; 4 Cancer Research Centre, The University of Edinburgh MRC Institute of Genetics and Molecular Medicine, Edinburgh, UK; 5 Department of Health Sciences, University of York, York, UK; 6 St. James’s University Hospital, Leeds Teaching Hospitals NHS Trust, Leeds, UK; 7 Academic Unit of Health Economics, University of Leeds Leeds Institute of Health Sciences, Leeds, UK; 8 Palliative Care, Leeds Teaching Hospitals NHS Trust, Leeds, UK; 9 Department of Oncology and Metabolism, The University of Sheffield, Sheffield, UK; 10 Psychological and Social Medicine, University of Leeds, Leeds, UK; 11 Leeds Cancer Centre, St James’s University Hospital, Leeds, UK

**Keywords:** ONCOLOGY, Protocols & guidelines, QUALITATIVE RESEARCH

## Abstract

**Introduction:**

In the UK National Health Service (NHS), most people with cancer are cared for at oncology outpatient services, where there are no standardised procedures for managing pain. As a result, patients with cancer may receive inadequate care for pain. The Cancer Pain-assessment Toolkit for Use in RoutinE oncology outpatient services aims to assess the feasibility of conducting a multicentre cluster-randomised trial of a systematic pain assessment and management programme integrated within routine care at UK NHS oncology outpatient services. This protocol describes an embedded process evaluation that aims to evaluate the acceptability, fidelity and implementation of the intervention and trial procedures.

**Methods and analysis:**

A combination of methods will be used in the process evaluation. Quantitative data on fidelity and intervention implementation will be collected using case report forms completed at sites, capturing details on training, intervention delivery and adherence. Qualitative data on acceptability and trial experience will be collected through semistructured interviews with intervention recipients (participants), intervention deliverers (healthcare professionals), research nurses and intervention champions. Researcher fieldnotes will also document trial acceptability throughout the trial. Quantitative data will be summarised descriptively. Qualitative data will be analysed using thematic analysis, guided by the framework of acceptability.

**Ethics and dissemination:**

The trial received ethical approval from South Yorkshire Research Ethics Committee and Health Research Authority (21/HRA/5245). Site-specific approvals were obtained from the research and innovation offices at Leeds Teaching Hospital and Hull Teaching Hospital. Trial findings will be disseminated through peer-reviewed publications and via participating sites.

**Trial registration number:**

ISRCTN86926298.

**STRENGTHS AND LIMITATIONS OF THIS STUDY**

- The process evaluation combines quantitative and qualitative methods to capture a comprehensive picture of the acceptability, fidelity and implementation of the intervention (Edinburgh Pain Assessment and Management Tool, EPAT+).
- The process evaluation involves diverse perspectives (eg, patients, staff and intervention champions) to assess acceptability, fidelity and implementation of the intervention (EPAT+).
- The process evaluation findings are context-specific and may not be generalisable to other settings and populations.

## Introduction

 In the UK, the prevalence of chronic pain among patients with cancer is estimated to be more than 70%.^1^ One-third of patients with cancer are undertreated for cancer pain,^2^ meaning they do not receive sufficient analgesia to manage their pain. Living with poorly managed cancer pain reduces patients’ quality of life,^3^ increases healthcare service use and costs^4^ and significantly reduces physical and emotional well-being.^5^ The burden of living with chronic cancer pain is also associated with a risk factor for anxiety and depression.^6^ Subsequently, uncontrolled pain is the most common reason for patients with cancer contacting general practitioner out of hours services.^7^ Numerous guidelines on managing cancer pain have been published in the last 25 years^1 8^; yet, in the UK, the majority of people with cancer are cared for at oncology outpatient services (OOS) where there are no standardised procedures for managing cancer pain.^8 9^ As a result, patients with cancer receive variable and inadequate care for pain.^3 10 11^

### The Cancer Pain-assessment Toolkit for Use in RoutinE oncology outpatient services study

The Cancer Pain-assessment Toolkit for Use in RoutinE oncology outpatient services (CAPTURE) study is a three-phase study funded by Yorkshire Cancer Research. First, we undertook a detailed qualitative evaluation of existing pain management processes at tertiary oncology referral centres in the UK.^9^ In phase two, we adapted the Edinburgh Pain Assessment and Management Tool (EPAT)^12^ for use in routine OOS, using a theoretically informed approach to complex intervention adaptation.^13 14^ The third phase is a multicentre cluster-randomised pilot trial. The trial aims to establish the feasibility of undertaking a definitive phase III multicentre cluster randomised trial within the UK National Health Service (NHS). This trial includes an embedded process evaluation which aims to evaluate the acceptability, fidelity and implementation of the intervention and trial procedures.

In its original form, EPAT^12^ was designed to prompt clinicians on oncology inpatient wards to systematically assess and manage cancer pain across the duration of a care episode. Pain scores were used to guide clinical decision-making and treatment using linked treatment algorithms. EPAT consists of four core components: (1) pain intensity screening, (2) detailed pain assessment, (3) treatment planning and (4) reassessment. These four core intervention components were designed to be integrated within existing routine care pathways on hospital oncology wards. Pain intensity screening (component one) was integrated within the patients’ vital-signs charts and completed hourly alongside other vital sign assessments (eg, blood pressure, temperature, pulse rate). If patients reported pain of 3 or greater (out of 10), this prompted a doctor or nurse to complete a detailed assessment of pain aetiology, mechanisms and impact (component two), resulting in a treatment plan (component three). Pain intensity and response to treatment were monitored as part of the vital-signs hourly reassessment (component four). To facilitate the implementation of EPAT within ‘normal practice’ (ie, support behaviour change), EPAT was integrated within ward policy and standard operating procedures, and all staff working on EPAT wards were trained in its use. Standard operating procedures and training materials described healthcare professionals (HCPs) undertaking intervention components as ‘intervention deliverers’ and patients with cancer reporting pain as ‘intervention receivers’. Additionally, an EPAT champion was identified at each ward to facilitate staff training, monitor EPAT use (ie, fidelity) and deliver additional ‘top-up’ training where necessary.

In order for EPAT to fit within the new context (oncology outpatient settings), we undertook a theoretically informed systematic adaptation of EPAT.^15^ We undertook a series of qualitative interviews (phase 1). We conducted codesign workshops with oncology outpatient HCPs and systematically deconstructed the intervention, identified the core and peripheral components and reconstructed the intervention to fit within oncology outpatient settings (phase 2).^15^ The adaptation process was supported by three expert panels: (1) experts by lived experience, (2) experts by clinical experience and (3) experts by academic experience who cofacilitated the workshops and supported the research team to evaluate and integrate workshop outcomes into the adapted version of EPAT, which is subsequently referred to as EPAT+.

The final prototype intervention (EPAT+) was adapted to support pain management for individuals living with cancer who are being cared for at OOS; EPAT+addresses treatment-related cancer, tumour-related cancer pain, as well as non-malignant causes of chronic pain in patients with cancer.^15^ An outline of the core components of EPAT+ and the modifications made to the intervention are published elsewhere.^15^

### The CAPTURE pilot trial process evaluation

The final phase of the CAPTURE study is a multicentre cluster randomised pilot trial. 12 outpatient services from 2 NHS tertiary oncology referral centres in the North of England will be randomly allocated (1:1) to deliver EPAT+in addition to usual care or usual care alone. It is anticipated that usual care will consist of appropriate individual pain assessment by nursing and medical staff, followed by a management decision. At present in the UK, this part of cancer care is not carried out in a structured, systematic fashion. While pharmacological management is based on the principles of WHO guidelines, the way in which these guidelines are used is not standardised.

A formal power calculation is not required for this feasibility study, as it is not designed to estimate effectiveness. Teare *et al* recommend^16^ that external feasibility trials include at least 60 participants per arm when the primary outcome is binary. Allowing for a 30% loss to follow-up and rounding to ensure balance across clinics (15 participants per clinic across 12 clinics), a total sample size of 180 participants (90 per arm) is deemed sufficient to meet the study objectives. Thus, 180 eligible patients will be recruited. An embedded process evaluation will assess acceptability, fidelity and intervention implementation. Process evaluations offer insights into implementation variation, resource use, participant roles, contextual factors and their impact on outcomes.^17^ Pilot trials commonly evaluate feasibility, acceptability and fidelity of the trial methods and intervention, aligning with Medical Research Council (MRC) guidance.^14 18^ This helps researchers to systematically evaluate intervention design, address practical challenges and understand acceptability to participants and those delivering the intervention, supporting refinements before a larger trial.

Assessing intervention acceptability has become essential in healthcare design and implementation due to the involvement of multiple intervention components for both deliverers (eg, HCPs) and recipients (eg, participants).^18 19^ A theoretical acceptability framework explores intervention acceptability across seven components: affective attitude, burden, perceived effectiveness, ethicality, intervention coherence, opportunity costs and self-efficacy.^19^ The CAPTURE pilot trial will enable the acceptability of EPAT+ to be explored on a large scale, with intervention components evaluated by both those that deliver them and those that receive them.

Intervention fidelity refers to how closely an intervention is implemented as intended, potentially moderating the relationship between the intervention and its outcomes.^20 21^ It includes measurable quantitative and qualitative aspects across five dimensions: adherence, dosage, quality of delivery, participant responsiveness and programme differentiation. Assessing fidelity across these levels enhances fidelity itself, supporting both the internal and external validity of larger trials.

### Aims and objectives

This protocol paper will focus specifically on the embedded process evaluation. There will be a separate paper that presents the protocol for the multicluster randomised pilot trial. This process evaluation has two aims: (1) to assess fidelity of intervention component delivery within OOS settings and (2) to understand the acceptability of the intervention components and trial processes to those delivering and receiving the intervention. These two aims address the implementation and contextual factors described in the MRC framework for process evaluation of complex interventions.^18^

The specific objectives for each aim are:

1. Fidelity: establish the fidelity of intervention component delivery across five core dimensions of fidelity, guided by An *et al*^21^:
  1. Training: To evaluate the adequacy of HCP training for delivery of the intervention.
  2. Adherence: To establish the extent to which each intervention component was delivered as planned.
  3. Dosage: To establish the amount (frequency) of intervention exposure.
  4. Reach: To establish the extent to which all eligible patients were exposed to EPAT+.
  5. Quality of delivery: To evaluate the quality of HCPs’ interaction with intervention components (the way the interventionist delivers the intervention using the overall process and strategies as prescribed by the developer).
  6. Participant responsiveness (Enactment): To understand the degree to which HCPs and participants respond to the intervention components (indicates the extent to which participants respond to or are engaged by the intervention components).
2. Acceptability: Establish the acceptability of the intervention and trial experience using the Framework of Acceptability^19^:
  1. Assess the acceptability of each intervention component and explore contextual factors associated with variation in acceptability, which may give an indication of future uptake.
  2. Explore the barriers and facilitators to the trial procedures, recruitment activities, retention and influential contextual factors from the perspective of research nurses (RNs).
  3. Explore the experiences of trial participation from the perspective of participants and HCPs.

## Methods

### Cluster trial design

A multicentre, two-arm, pilot cluster randomised controlled trial, comparing EPAT+ (intervention) with usual care from at least two NHS tertiary oncology referral centres (sites) in the North of England. The cluster pilot trial protocol is described in detail elsewhere^22^; however, in brief, the structure of the trial clusters has been designed to capture variation in oncology outpatient clinics by centre type (eg, tertiary referral centre, district general hospital), staffing background (eg, oncologist, nurse, clinical support worker) and clinic process nature (eg, single or multiple consultants-led clinics).

Eligible patients approached regarding study participation, and those consenting will complete three follow-ups, at 1 week, 1 month and 2 months. Data collected from a mix of patient-reported questionnaires (available online or paper formats), and data extracted from electronic health records.

### Process evaluation design

We will use qualitative and quantitative methods to address the process evaluation aims, embedded within the CAPTURE pilot trial. The process evaluation will involve assessment with four participant groups:

1. Patient-participants of the CAPTURE pilot trial (ie, recipients of the intervention).
2. HCPs working in OOS (ie, oncologists, clinical support workers, nurses) during the trial and delivering the EPAT+intervention (ie, intervention deliverers).
3. Intervention champions (HCPs at site that lead implementation of the intervention).
4. RNs involved in trial research activities.

Patient-participants, RNs and HCPs will be recruited from both trial arms.

The process evaluation aims will be delivered by capturing data for each objective in the following way:

#### Fidelity: training

The number of training sessions and number of role-play sessions delivered to HCPs and interventions champions will be recorded, in addition to any refresher or follow-up training required throughout the duration of the trial. The overall number of HCPs trained to deliver the intervention will be recorded.

#### Fidelity: adherence

The number of patient-participants in the intervention arm who receive pain screening (EPAT+step 1) and the number of patients with a pain score ≥3/10 for whom EPAT+step 2 is completed will be recorded. The number of patient-participants receiving each element of EPAT+step 2 (eg, an onward referral to a pain specialist) and the number of self-management booklets provided to patients will be recorded.

#### Fidelity: dosage and reach

The number of EPAT+forms completed each time the patient-participants come into contact with EPAT+at recurring outpatient appointments, and the number of EPAT+forms completed versus total number of people on the OOS clinic list.

#### Fidelity: quality of delivery

The experiences and perspectives of HCPs from the qualitative interviews will explore the extent to which they felt they were able to use the intervention materials and how they believed they were able to deliver the intervention as it was intended. This will build on the quantitative data collected to inform aims 1b and 1c.

#### Fidelity: participant responsiveness

Patient-participants’ experiences and perspectives of receiving (ie, how well they believed HCPs discussed pain, did they receive the self-management materials) EPAT+intervention will explore the extent to which they felt they had engaged with the intervention.

#### Acceptability of the intervention components

The assessment of acceptability of the intervention will be guided by the TFA. Acceptability will be assessed at the end of the trial, through semistructured interviews with patient-participants and HCPs.

#### Barriers and facilitators/trial experience

Trial experience will be assessed using qualitative semistructured interviews and researcher (OCR) fieldnotes taken during and after the trial. Interview questions will be informed by the framework of acceptability. The interview will explore barriers and facilitators to trial procedures, recruitment and experience of being part of the trial.

### Patient and public involvement and engagement

At the project’s outset, we established a patient and public involvement and engagement (PPIE) group, comprising individuals with personal experience of managing cancer pain and a former caregiver. One PPIE member also joined the research team as a grant coapplicant. The group met during the study development phase and contributed to the design and proposed trial delivery methods.

### Sampling and recruitment

#### Patient-participants (intervention recipients)

Patient-participants are individuals who have a diagnosis of cancer; attend an OOS; are aged 18 years or over; are not considered by their clinical team to be too ill to take part or actively dying; and can self-report a score of ≥3 on the 0–10 Numerical Rating Scale for worst pain (including common pain descriptors such as aching, unpleasant, niggling, discomfort, dull ache, cramp, throb, pinch, sharp, sting) in the past 72 hours, in any part of their body.

Patient-participants will be eligible for participation in the qualitative interview if: (1) they were recruited to provide outcome data for the trial; ((2) they provided baseline trial data and ((3) they are willing to participate in a one-off interview about their experiences of receiving EPAT+during consultations and of the trial processes. Patient-participants will not be eligible for participation in an interview if they decided to withdraw from the trial at any point. Participation in the end of trial interview is optional. When providing initial consent at baseline, participants will be asked if they are willing to be contacted by a member of the research team at the end of the trial for an interview. If willing, participants will be contacted after the completion of their 2-month follow-up questionnaire by telephone. Written or telephone consent will be obtained. Purposive sampling will be used to identify a total of 10 (5 per arm) participants from both intervention and usual care services to take part in an end of trial interview.

#### HCPs (intervention delivers)

HCPs (ie, doctors, healthcare assistants, nurses) from OOS that were part of the trial will be invited to participate in an end of trial interview about their experiences. HCPs will be eligible for participation if: (1) they worked at a participating OOS during the study period and (2) they were recruited from the intervention arm of the trial and have experience of EPAT+or if recruited from the usual care arm of the trial, they have experience of trial processes. This could include HCPs with varied experience, for example, consultants, registrars, healthcare assistants. Across all intervention OOS, a minimum of four HCPs will be recruited (one HCP per service). However, where there is more than one HCP involved in the delivery of EPAT+at a service, all will be invited to participate in the interview. At the end of the trial, HCPs will be approached by the research fellow (OCR) via email with an information pack that includes an invitation letter, information sheet and consent form. If interested, written or telephone consent will be obtained.

#### Intervention champions

OOS randomised to the intervention arm identified intervention champions to lead the implementation of the intervention, with support from the OOS clinical team. Intervention champions will be required to (1) have oversight of the outpatient service randomised to the intervention arm; (2) have direct interaction with patients and their medical notes; (3) have direct contact with clinical staff responsible for pain management and (4) experience of rolling out new staff processes (desirable). At the end of the trial, they will be approached to take part in an interview by the research fellow (OCR) via email with an information pack that includes an invitation letter, information sheet and consent form. If interested, written or telephone consent will be obtained. Up to four intervention champions will be interviewed.

#### RNs in OOS

Across all OOS, a minimum of four RNs who were responsible for carrying out the trial and research-related activities will be recruited to take part in an end of study interview. However, where there were more RNs involved in the study at an OOS, all will be invited to participate in an interview. At the end of the trial, they will be approached by the research fellow via email with an information pack that includes an invitation letter, information sheet and consent form. If interested, written or telephone consent will be obtained.

### Data collection: qualitative interviews overview

Semistructured interviews will be conducted to assess fidelity and quality of intervention implementation and identify the contextual factors that are associated with any variation in intervention uptake, outcome measures and trial processes. Interviews will be conducted with patient-participants (from both intervention and usual care arms), intervention champions and HCPs (from both intervention and usual care arms) in OOS and RNs that were involved in trial research activities. Interviews will last up to 1 hour.

**Figure 1:**
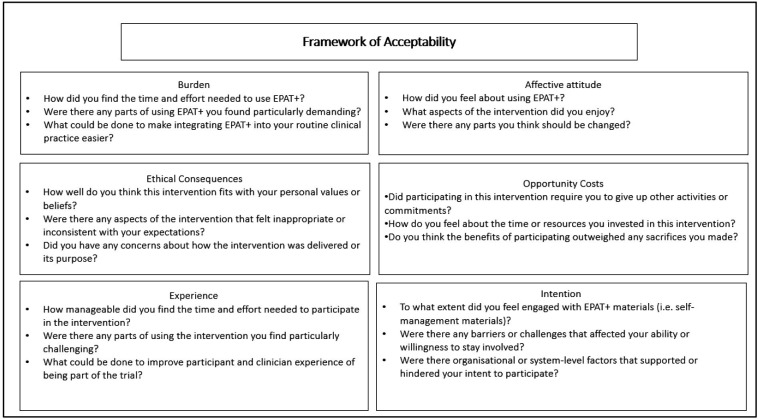
Adapted framework of acceptability. EPAT**,** Edinburgh Pain Assessment and management Tool.

Interview topic guides for participant groups have been developed by the research team in line with the framework of acceptability^19^ and previous literature.^12^ An adapted framework of acceptability will guide the questions used to explore the acceptability of EPAT+ (figure 1). Interviews will be conducted via telephone, videoconferencing software (eg, Microsoft Teams) or in-person at site. Interviews will be recorded using either an audio recorder (in case of telephone or in-person interviews) or using the recording function via videoconferencing software. All video or audio files of interviews will be deleted once the interview has been transcribed verbatim and analysed (in case recordings need to be returned to during analysis to check meaning or accuracy). Transcription and analysis will be done by the University of Leeds research team.

#### Qualitative interviews with patient-participants (intervention receivers)

Interviews will assess patient-participants’ experiences of being part of the trial, burden related to trial processes (ie, completion of trial questionnaire, length of follow-up time points) and affective attitude towards the intervention (ie, review of self-management materials, EPAT+form). Interview topic guides for patient-participants have been reviewed and informed by the PPIE group. Data will be used to inform the data collection strategy for a larger, definitive trial.

#### Qualitative interviews with HCPs (intervention delivers)

Interviews will assess uptake and fidelity of the intervention within OOS, contamination, acceptability of trial procedure and adherence. They will also explore changes in clinicians’ pain assessment practices or patients’ pain control (eg, access to self-management resources, tailored analgesic prescribing).

#### Qualitative interviews with intervention champions

Interviews will assess uptake and fidelity of the intervention, acceptability of trial procedures and training materials of the intervention itself and their experiences regarding any interactions they have had with patient-participants. This includes recruitment and consent procedures, completion of outcome questionnaires with patients at baseline and trial-related case report forms (CRFs) and future areas for improvements if a definitive trial is conducted. We will also explore suggested improvements to the intervention components and training materials to establish if it would be feasible to implement the intervention into routine practice in the NHS.

#### Qualitative interviews with RNs

Interviews will assess the uptake and acceptability of trial procedures. This includes recruitment and consent procedures, completion of outcome questionnaires with patients at baseline and trial-related CRFs and future areas for improvements if a definitive trial is conducted.

#### Researcher fieldnotes

The research fellow (OCR) will maintain informal fieldnotes throughout the trial, documenting observations on barriers and facilitators to intervention implementation and the acceptability of trial processes. These handwritten notes will be expanded and typed into more detailed records.

### Data analysis

#### Qualitative analysis

Thematic analysis will be conducted in six stages: familiarisation, coding, generating themes, reviewing and defining themes and writing up.^23 24^ This analysis will identify barriers and facilitators to implementing EPAT, challenges with intervention procedures, outcome measures and areas for improvement. Themes from interviews and researcher fieldnotes will capture participant feedback, helping to understand differences in intervention acceptability.

NVivo will be used for data management, with verbatim quotes incorporated to substantiate findings. OCR will lead the analysis, while MRM and SP will perform secondary coding. To ensure inter-rater reliability, a second researcher will code 10% of the transcripts. Regular team meetings, including coapplicants and PPIE members, will be held to discuss and resolve any coding discrepancies, either by two researchers or, if needed, by the entire team. Codes will be organised into a thematic framework to compare participant perspectives and inform insights into trial experiences and intervention uptake.

#### Quantitative analysis

Quantitative data related to fidelity of intervention delivery (training, adherence, dosage and reach) will be captured via study CRFs completed at each site by the RN staff. Quantitative data will be summarised descriptively:

- Categorical data will be summarised as number and proportion overall, by trial arm and by OOS.
- Continuous data will be summarised as means (SD) overall, by trial arm and by OOS.

## Ethics and dissemination

The CAPTURE pilot trial received research ethical approval from South Yorkshire Research Ethics Committee and Health Research Authority (21/HRA/5245) and site-specific approval was obtained from the research and innovation offices at Leeds Teaching Hospital and Hull Teaching Hospital. The CAPTURE pilot trial is registered on the ISRCTN registry (86926298). Trial findings will be disseminated through peer-review publications. All trial data will be securely archived at the University of Leeds for a minimum of 5 years.

### Trial status

The study opened for recruitment on 4 December 2023 and was open at the time of submission.
