# Supplementary material for: Acceptability, fidelity and implementation of systematic integrated pain management in oncology outpatient services: a process evaluation protocol for a multicentre clustered randomised pilot trial
Source: BMJ Open. 2025 Jun 27;15(6):e101935. doi: 10.1136/bmjopen-2025-101935 (PMC12207156; doi:10.1136/bmjopen-2025-101935)
Supplement: Supplementary file 1 [file bmjopen-2025-101935.reviewer_comments.pdf]

PEER REVIEW HISTORY

BMJ Open publishes all reviews undertaken for accepted manuscripts. Reviewers are asked to complete a checklist review form and are provided with free text boxes to elaborate on their assessment. These free text comments are reproduced below.

ARTICLE DETAILS

Title (Provisional)

Acceptability, fidelity and implementation of systematic integrated pain management in Oncology Outpatient Services: A Process evaluation protocol for a multi-centre clustered randomised pilot trial (ISRCRN86926298)

Authors

Robinson, Olivia Claire; Day, Florence; Boland, Elaine G; Collinson, Michelle; Fallon, Marie; Farrin, Amanda; Flemming, Kate; Girvan, Sean; Hartup, Sue; Meads, David; Hurlow, Adam; Mayland, C; O'Dwyer, John; Pini, Simon; Swinson, Daniel; Richards, Suzanne H; Mulvey, Matthew R.

VERSION 1 - REVIEW

Reviewer 1

Name Ortiz-Comino, Lucía

Affiliation Faculty of Health Sciences (Melilla), Department of Physical Therapy, University of Granada, Melilla, Spain, Physiotherapy

Date 06-Apr-2025

COI None

This manuscript is of interest as it addresses pain perceived by oncology outpatients, which as authors state in their introduction, a third of cancer patients are under-treated for this symptom.

In general, manuscript is well written and objectives and aims are clearly described. Also, the statistical analysis is well defined.

I would only suggest the authors to include a brief discussion where they could expose the strengths and limitations of this study, as well as the possible clinical implications of their study.

Moreover, as the journal indicates, I would reccomend authors to include the SPIRIT checklist in their supplemental material.

Reviewer 2

Name Liu, Lingxiang

Affiliation Jiangsu Province People's Hospital and Nanjing Medical University First Affiliated Hospital, Department of Oncology

Date 02-May-2025

COI None

The MS. is about to use the systematic pain management protocol (EPAT+) to the oncology outpatient setting, and utilizing a theory-driven multidimensional process evaluation to provide scientific evidence. Some points may be paid attention as follows.

1. Would you please describe the details, core components, and key modifications of EPAT+?

2. The definition of "usual care" for the control group is ambiguous. Variations in usual care may introduce significant heterogeneity, complicating the interpretation of results.

3. How to control numerous uncontrollable confounding variables, such as the multi-center, multi-staff, and multi-process nature of the study?

4. Would please give the details for potential risks and adverse event management, and safeguards for data security and privacy?

5. How about an expected sample size of 180 patients, would you please provide the formula, assumed parameters, or statistical power calculations?

6. How to plans for confounding factors, how to take about the stratified randomization across centers, and adjustments for center effects during analysis?

VERSION 1 - AUTHOR RESPONSE

	Reviewer 1	
1	I would only suggest the authors to include a brief discussion where they could expose the strengths and limitations of this study, as well as the possible clinical implications of their study.<br>Moreover, as the journal indicates, I would recommend authors to include the SPIRIT checklist in their supplemental material.	Thank you for your recommendation to include a spirit checklist. As requested by the editor (comment 8) we have provided a CONSORT extension as supplementary material.
	Reviewer 2	
2	Would you please describe the details, core components, and key modifications of EPAT+?	Thank you for highlighting this. We have included additional information in the introduction to make it clearer that there is already a published article that outlines the core components and key modifications of EPAT+.

3	The definition of "usual care" for the control group is ambiguous. Variations in usual care may introduce significant heterogeneity, complicating the interpretation of results.	We have included further information on the ‘usual’ care group in the capture process evaluation section.
4	How to control numerous uncontrollable confounding variables, such as the multi-center, multi-staff, and multi-process nature of the study?	We have included additional information on the confounding variables included in the pilot trial. However further detail of this information will be in our pilot trial protocol paper that is currently under-review with pilot and feasibility studies.
5	Would please give the details for potential risks and adverse event management, and safeguards for data security and privacy?	The risks and adverse events are presented in our pilot-trial protocol paper. Therefore, we have not responded directly to this question, as we it will not be included in the process evaluation.
6	How about an expected sample size of 180 patients, would you please provide the formula, assumed parameters, or statistical power calculations?	A formal power calculation is not required for this feasibility study, as it is not designed to estimate effectiveness. However, we have included additional rationale for the 180-sample size.
7	How to plans for confounding factors, how to take about the stratified randomization across centers, and adjustments for center effects during analysis?	The detailed statistical analysis plan is presented in our pilot-trial protocol paper which includes plans for dealing with confounding and variation across centres. We have not responded directly to this question here, as we will not be including the analysis of these data within the process evaluation.
